# A new meroterpenoid functions as an anti-tumor agent in hepatoma cells by downregulating mTOR activation and inhibiting EMT

**DOI:** 10.1038/s41598-018-31409-2

**Published:** 2018-09-03

**Authors:** Haoqiang Wan, Jiemei Li, Keda Zhang, Xiaoting Zou, Lanlan Ge, Fuqiang Zhu, Huirong Zhou, Minna Gong, Tianwa Wang, Dongling Chen, Shusong Peng, Boping Zhou, Xiaobin Zeng

**Affiliations:** 1 Center Lab of Longhua Branch, Shenzhen People’s Hospital, 2nd Clinical Medical College of Jinan University, Shenzhen, 518120 Guangdong Province China; 2 Department of Infectious disease, Shenzhen People’s Hospital, 2nd Clinical Medical College of Jinan University, Shenzhen, 518120 Guangdong Province China; 3 Department of pathology (Longhua Branch), Shenzhen People’s Hospital, 2nd Clinical Medical College of Jinan University, Shenzhen, 518120 Guangdong Province China

## Abstract

Liver cancer, also known as primary liver cancer, is cancer that starts in the liver. JNU-144, a new meroterpenoid purified from *Lithospermum erythrorhizon*, has exhibited promising anticancer activity; however, the molecular mechanisms of action of JNU-144 on malignant cells remain unclear. Our studies revealed that JNU-144 suppressed cell viability and proliferation in hepatoma cells by downregulating mTOR activation. Meanwhile, JNU-144 activated the intrinsic apoptosis pathway and subsequently triggered apoptotic cell death in SMMC-7721 cells. We also found that JNU-144 inhibited the epithelial–mesenchymal transition in both SMMC-7721 and HepG2 cells through reprogramming of epithelial–mesenchymal transition (EMT)-related gene expression or regulating protein instability. These findings indicate that JNU-144 exerts potent anticancer activity in hepatoma cells and may be developed as a potential therapeutic drug.

## Introduction

Cancer that begins in the liver is referred to as primary liver cancer^1^. Correspondingly, cancer that spreads from other tissues to the liver is known as liver metastasis, which is much more common^2^. Hepatocellular carcinoma (HCC), formed by malignant hepatocytes, accounts for approximately 75% of all cases of primary liver cancer^3^. The main cause of liver cancer is cirrhosis of the liver caused by hepatitis B, hepatitis C or alcohol^4^. Other risk factors include aflatoxin, non-alcoholic fatty liver disease and liver flukes. As of 2010, primary liver cancer resulted in 754,000 deaths globally, making it the third-most lethal form of cancer^5^. Primary liver cancer shows a conspicuous geographical and sexual distribution. Southeast Asia and the west coast of Africa are the most high-risk areas^6^. Males are more affected by HCC than females^2^. While five-year survival rates after resection have dramatically improved over the last few decades, subsequent recurrence can exceed 70%^7^, which is a huge challenge facing researchers.

Metastasis is largely responsible for the recurrence, poor prognosis and death of cancer. The initial stage of the metastatic process is dependent on the epithelial–mesenchymal transition (EMT). During EMT, cells undergo a fundamental change in cellular morphology and increase their ability to migrate^8^. Upon the initiation of EMT, epithelial cell–cell junctions are deconstructed and the junction proteins are degraded or re-localised^9^. As EMT plays a crucial role in cell differentiation and metastasis in cancer progression, it is tightly regulated through cooperation and crosstalk between signaling pathways *in vivo*^10,11^. With the increased understanding of the regulatory networks defining EMT, the search for specific inhibitors of tumor metastasis has become even more promising.

**Figure 1:**
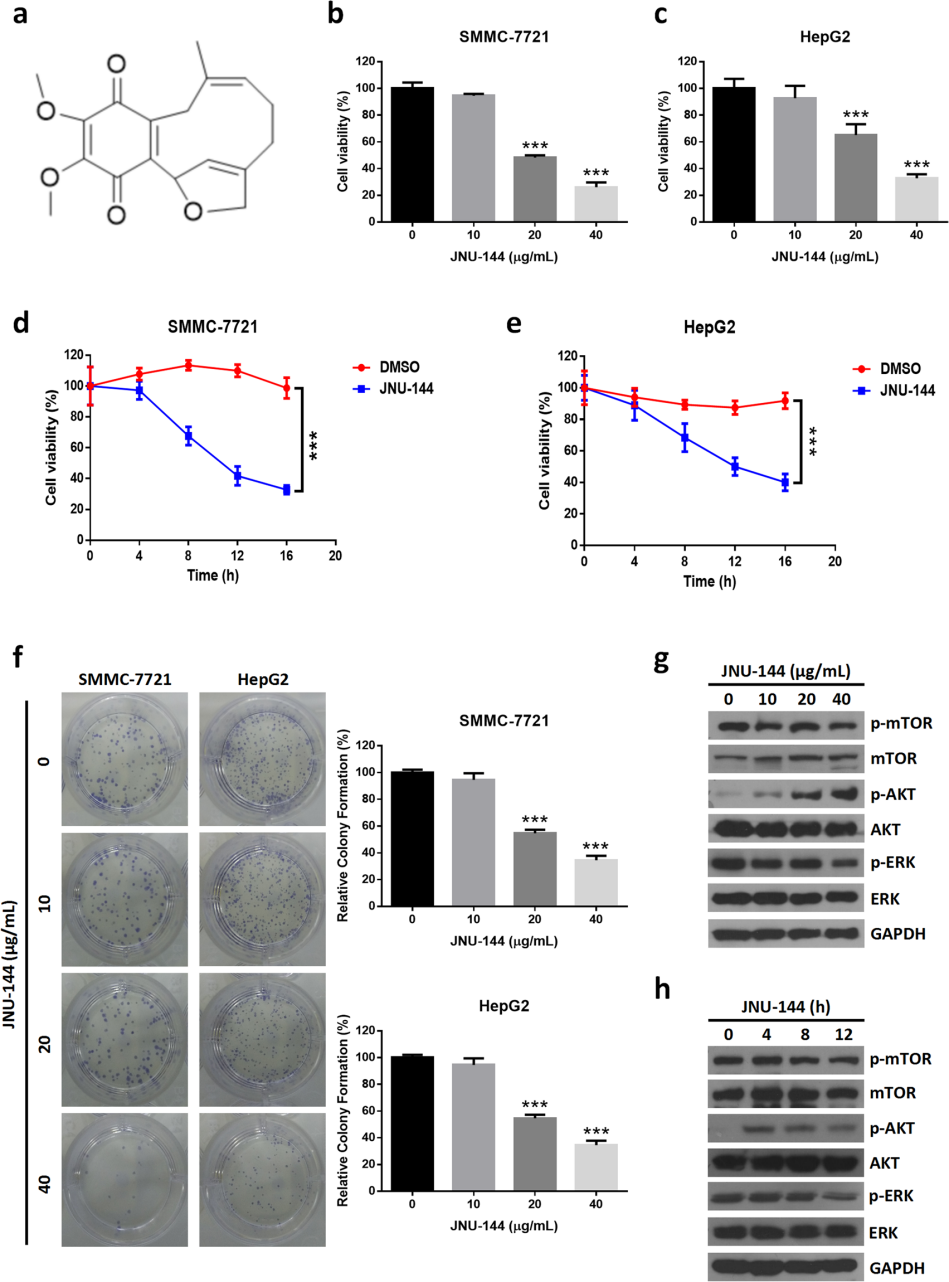
JNU-144 inhibits cell viability and proliferation in hepatoma cells by downregulating activation of mTOR. (**a**) The chemical structure of JNU-144. SMMC-7721 (**b**) and HepG2 (**c**) cells were exposed to various concentrations of JNU-144 for 12 h for the MTT assays to evaluate the cell viability. SMMC-7721 (**d**) and HepG2 (**e**) cells were exposed to JNU-144 at the concentration of 20 μg/mL for indicated time for the MTT assays to evaluate the cell viability. (**f**) Colony formation assays were performed with SMMC-7721 and HepG2 cells stimulated with various concentrations of JNU-144 for 12 h to evaluate the cell proliferation. (**g**) SMMC-7721 cells treated with different concentrations of JNU-144 for 12 h were lysed and subjected to immunoblotting for detection of the expression levels of relative proteins. (**h**) SMMC-7721 cells treated with JNU-144 at the concentration of 20 μg/mL for indicated time were lysed and subjected to immunoblotting for detection of the expression level of relative proteins. ***p < 0.001 compared with the control group. Graphs show mean ± SD of triplicate wells and represent three independent experiments.

*Lithospermum erythrorhizon* is a plant belonging to the genus *Lithospermum*, which grows wild throughout China, Korea, and Japan^12^. The dried root of *L. erythrorhizon* is a traditional herbal medicine with various biological properties, including antibacterial^13^, anti-tumor^14^, anti-angiogenesis^15^ and antiviral^16,17^ activities. Our lab isolated and identified a new meroterpenoid JNU-144 from the dried root of *L. erythrorhizon* (Fig. 1a)^18^. In all hepatoma cell lines we examined, JNU-144 exerted potent anti-tumor effects. Specifically, in SMMC-7721 cells, JNU-144 induced apoptosis by activating the intrinsic apoptosis pathway. We also found that JNU-144 inhibited EMT in both SMMC-7721 and HepG2 cells by reprogramming the gene expression profile. Furthermore, JNU-144 suppressed tumor growth *in vivo*. These results suggest potential for JNU-144 as a novel therapeutic drug for liver cancer.

## Results

### JNU-144 inhibits cell viability and proliferation in hepatoma cells by downregulating mTOR activation

To investigate the effect of JNU-144 treatment on hepatoma cell viability, we performed an 3-(4,5-Dimethylthiazol-2-yl)-2,5-diphenyltetrazolium bromide (MTT) assay with SMMC-7721 and HepG2 cells. In both cell lines, JNU-144 treatment suppressed the cell viability in a dose- and time-dependent manner (Fig. 1b–e). Furthermore, we observed similar results in all other hepatoma cell lines we examined (Figure S1a–d). Then, we evaluated the effect of JNU-144 on cell proliferation using a colony formation assay. As expected, JNU-144 treatment resulted in a dose-dependent decrease in colony formation numbers in both SMMC-7721 and HepG2 cells (Fig. 1f). The PI3K/AKT/mTOR pathway is critical for cellular proliferation, growth, survival and mobility and is constitutively activated in many types of cancer^19^. Thus, we checked the activation of serine/threonine Kinase 1 (AKT) and mammalian target of rapamycin (mTOR). Surprisingly, treatment with JNU-144 decreased the levels of phosphorylated mTOR but significantly increased the levels of phosphorylated AKT in SMMC-7721 cells (Fig. 1g,h). Moreover, we observed similar results in HepG2 cells (Figure S1e,f). These data indicate that JNU-144 may block mTOR activation in an AKT independent pathway. As the MEK/ERK pathway functions as an important upstream regulator of mTOR signaling, we checked the phosphorylation of ERK1/2 after JNU-144 treatment in SMMC-7721 and HepG2 cells. We found that JNU-144 treatment inhibited ERK1/2 activation in a dose- and time-dependent manner (Fig. 1g,h and S1e,f). These findings were in accordance with a number of recent studies that have reported AKT independent regulation of mTOR signaling by the ERK/MAPK pathway^20^, which suggested that JNU-144 induced mTOR inhibition may be mediated by MEK/ERK pathway.

### JNU-144 triggers apoptosis through intrinsic pathway in hepatoma cells

**Figure 2:**
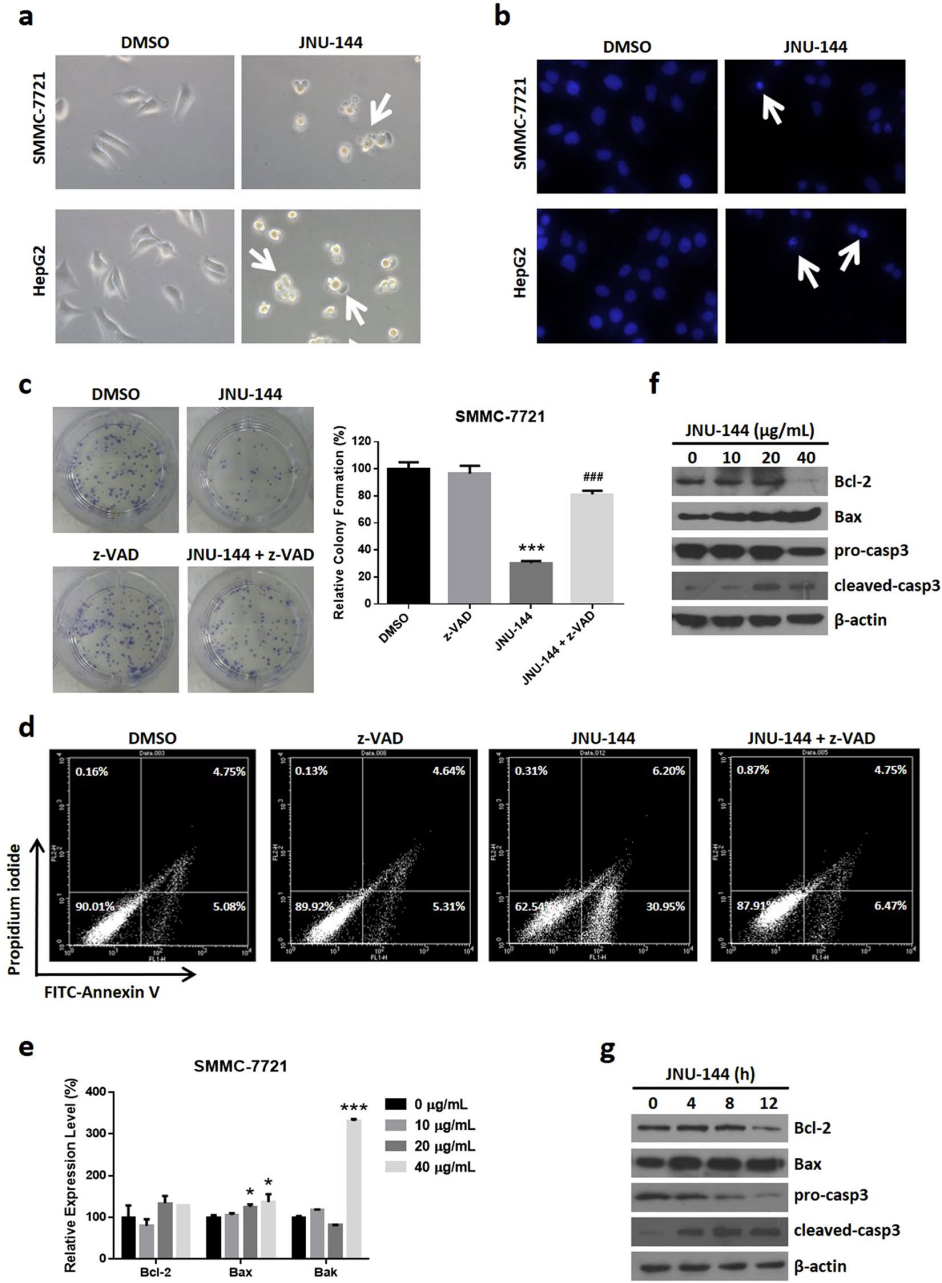
JNU-144 induces apoptosis in hepatoma cells. (**a**) SMMC-7721 and HepG2 cells stimulated with DMSO or 20 μg/mL JNU-144 for 12 h were photographed using a microscope. The arrows point to apoptotic cells. (**b**) SMMC-7721 and HepG2 cells stimulated with DMSO or 20 μg/mL JNU-144 for 12 h were stained with 0.1 μg/mL DAPI for 10 min, followed by photographed using a fluorescence microscope. The arrows point to cells with karyopyknosis. SMMC-7721 cells stimulated with DMSO or JNU-144 in the presence or abcence of z-VAD-fmk (z-VAD), a pan caspase inhibitor which is being widely used as an apoptosis inhibitor, were subjected to colony formation assay (**c**) and apoptosis assay (**d**). (**e**) Relative mRNA expression levels of intrinsic pathway related genes of SMMC-7721 cells stimulated with various concentrations of JNU-144 for 12 h was detected by real-time PCR. SMMC-7721 cells stimulated with various concentrations of JNU-144 for 12 h (**f**) or 20 μg/mL JNU-144 for different time (**g**) were lysed and subjected to immunoblotting for detection of the expression level of relative proteins. *p < 0.05 compared with the control group; ***p < 0.001 compared with the control group; ^**###**^p < 0.001 compared with the JNU-144 treated group. Graphs show mean ± SD of triplicate wells and represent three independent experiments.

We observed massive cell death after JNU-144 treatment. To confirm the type of cell death, we imaged cell morphology after treatment with JNU-144 or DMSO. We found it to be typical apoptotic cell death characterised by cell shrinkage, pyknosis, massive plasma membrane blebbing and the destruction of cell fragments into apoptotic bodies^21^. We observed extensive blebbing and pyknosis after 4–6-diamidino-2-phenylindole (DAPI) staining in JNU-144-treated cells by microscopy (Fig. 2a,b). The dependence of caspase activation is a major biochemical feature of apoptosis^22^. z-VAD-fmk is a pan caspase inhibitor, and it is widely used as an apoptosis inhibitor^23–25^. In our models, z-VAD-fmk is able to abolish the cell viability and proliferative suppression of JNU-144 treatment in SMMC-7721 (Fig. 2c) and HepG2 (Figure S2a) cells. We further performed the apoptosis assay by Annexin V/Propidium Iodide staining. As shown in Fig. 2d and S2b, JNU-144 treatment increased the percentage of apoptotic cells, which is partially inhibited by caspase inhibitor z-VAD-fmk. In summary, JNU-144 treatment induced apoptosis in hepatoma cells. To validate the apoptosis-inducing mechanism of JNU-144, we assessed the mRNA levels of several regulators of apoptosis, including anti-apoptotic protein B-cell lymphoma-2 (bcl-2) and pro-apoptotic proteins bcl-2 associated X protein (bax) and bcl-2 antagonist killer (bak). The mRNA level of bak was increased significantly while the mRNA levels of bax and bcl-2 showed no remarkable changes (Fig. 2e). Furthermore, the western blotting assay showed that JNU-144 induced the expression of bax and reduced the expression of bcl-2 (Fig. 2f,g and S2c), which is consistent with our results about mRNA mentioned above. Moreover, we observed the cleavage of pro-caspase 3 following JNU-144 treatment in SMMC-7721 (Fig. 2f,g) and HepG2 (Figure S2c) cells. Taken together, these data suggest that JNU-144 induces the intrinsic pathway and triggers apoptosis in hepatoma cells.

### JNU-144 suppresses EMT in hepatoma cells

**Figure 3:**
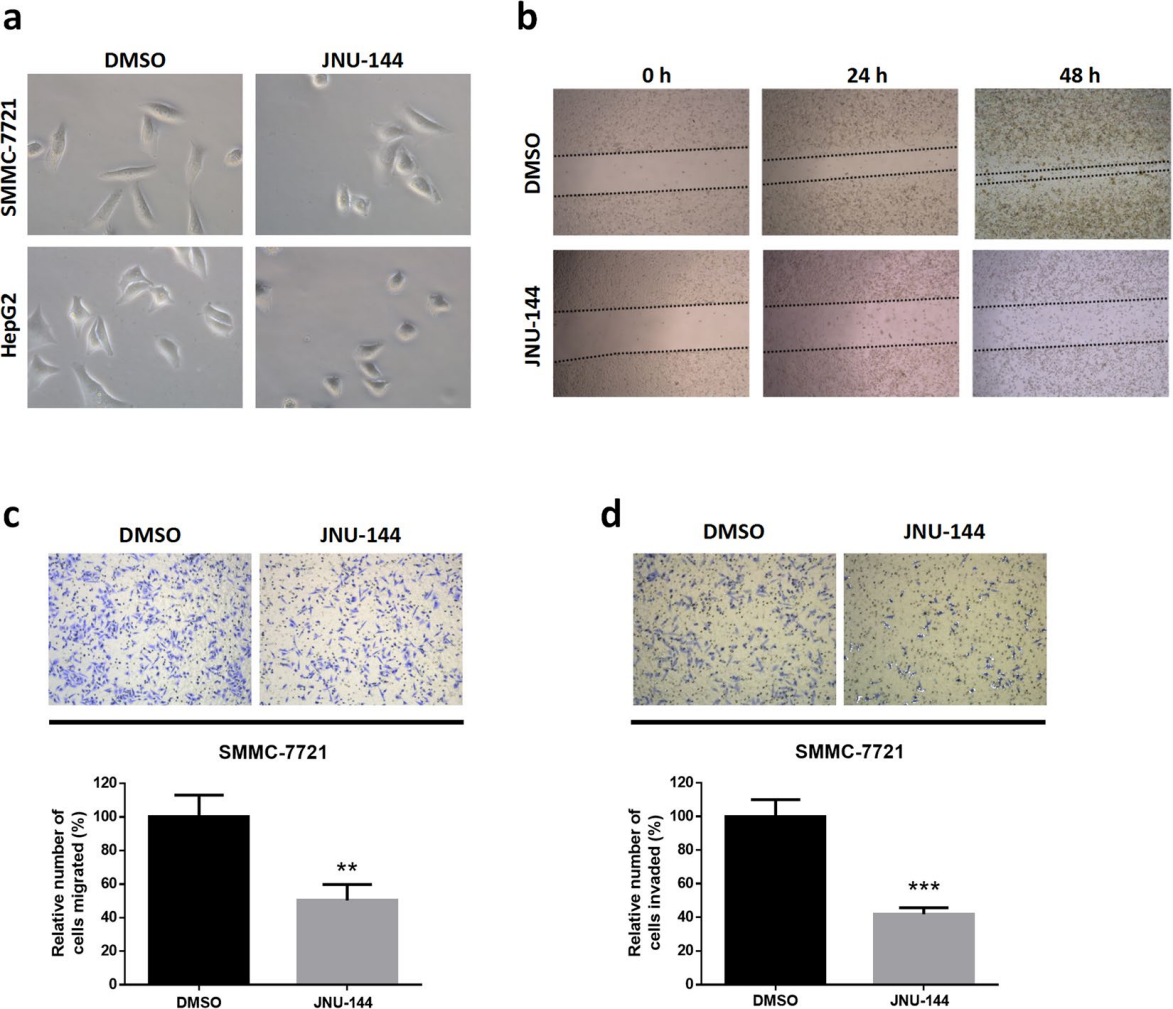
JNU-144 inhibits EMT in hepatoma cells. (**a**) SMMC-7721 and HepG2 cells stimulated with DMSO or 10 μg/mL JNU-144 for 12 h were photographed using a microscope. (**b**) SMMC-7721 cells were pretreated with DMSO or 10 μg/mL JNU-144 for 12 h, followed by scraping with a pipette tip. The wounded area was photographed after scraping for 0, 24 and 48 h. SMMC-7721 cells pretreated with DMSO or 10 μg/mL JNU-144 for 12 h were used for *in vitro* migration (**c**) or invasion (**d**) assays. **p < 0.01 compared with the control group; ***p < 0.001 compared with the control group. Graphs show mean ± SD of triplicate wells and represent three independent experiments.

EMT is a biologic process that allows the polarised epithelial cell to undergo multiple biochemical changes and obtain the mesenchymal cell phenotype, including elevated migratory capacity and invasiveness^26^. In consideration of the important role that EMT plays in tumor cell migration and metastasis, we wondered whether JNU-144 might have effects on EMT in hepatoma cells. We observed JNU-144 induced dramatic morphological changes, from fibroblastoid spindle-shaped cells to compact, cobblestone-like epithelial structures (Fig. 3a). The effect of JNU-144 on the migration of hepatoma cells was evaluated with an *in vitro* wound-healing assay and transwell assay. JNU-144 significantly suppressed the migration of SMMC-7721 (Fig. 3b,c) and HepG2 cells (Figure S3a,b). To assess the effect of JNU-144 on the invasion of hepatoma cells, we conducted a transwell assay using matrigel-coated chambers. Compared to the negative control, JNU-144 treatment significantly decreased the number of penetrated SMMC-7721 (Fig. 3d) and HepG2 cells (Figure S3c). These results suggest that JNU-144 exerts potent inhibitory effects on the migration and invasiveness of hepatoma cells *in vitro*.

### JNU-144 inhibits EMT through reprogramming of EMT-related gene expression

**Figure 4:**
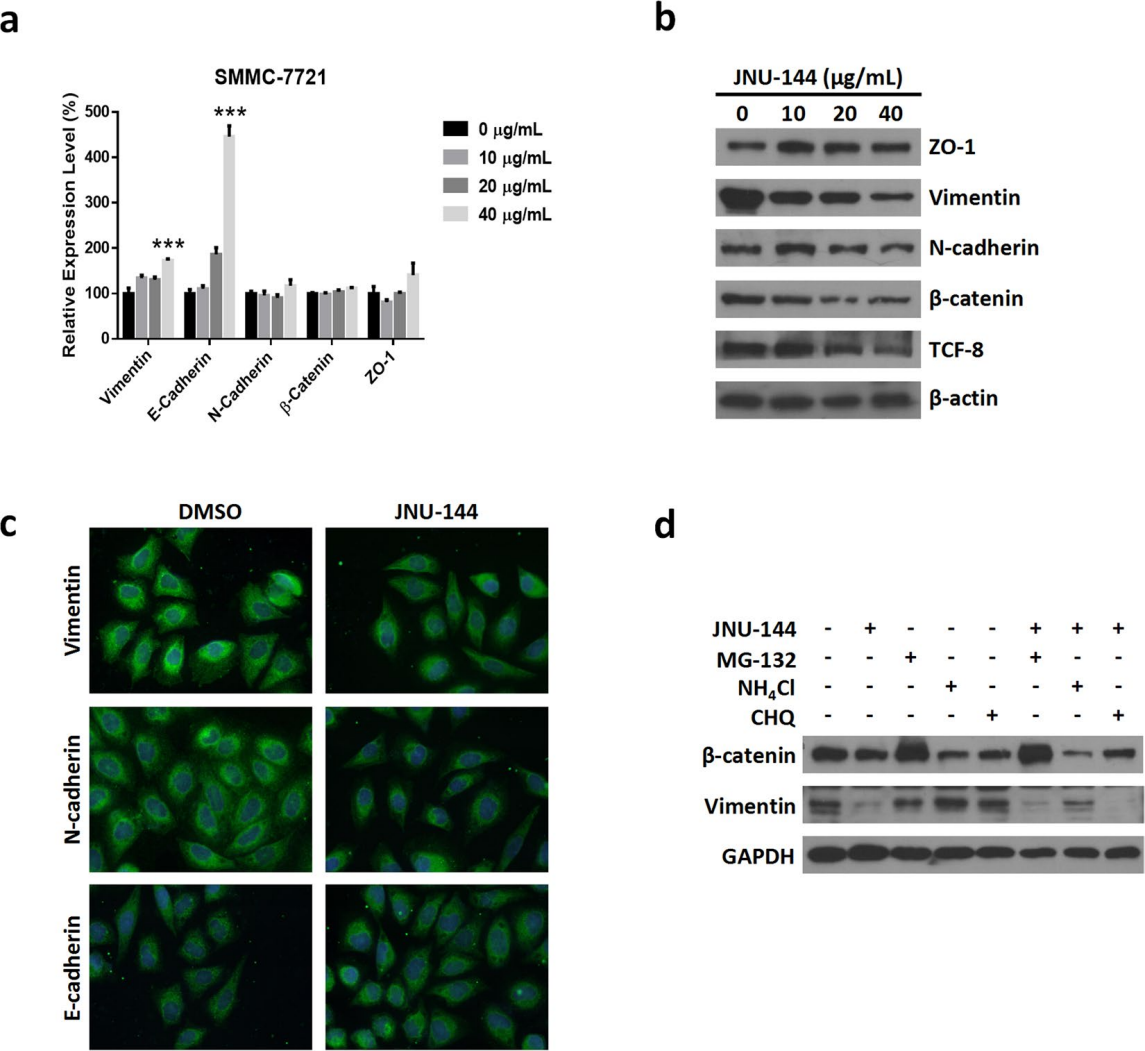
JNU-144 reprogrammes EMT related gene expression profile. (**a**) Relative mRNA expression level of EMT related genes of SMMC-7721 cells stimulated with various concentrations of JNU-144 for 12 h was detected by real-time PCR. (**b**) SMMC-7721 cells stimulated with various concentrations of JNU-144 for 12 h were lysed and subjected to immunoblotting for detection of the expression level of relative proteins. (**c**) SMMC-7721 cells stimulated with DMSO or 10 μg/mL JNU-144 for 12 h were immunostained and photographed using a fluorescence microscope. (**d**) SMMC-7721 cells were pretreated with proteasome inhibitor MG-132 (20 μM), lysosome inhibitor ammonium chloride (15 mM) or chloroquine (100 μM) for 12 h, followed by stimulation with DMSO or 20 μg/mL JNU-144 for 12 h. The cells were lysed and subjected to immunoblotting for detection of the expression level of relative proteins. ***p < 0.001 compared with the control group. Graphs show mean ± SD of triplicate wells and represent three independent experiments.

To define the mechanism that underlies the inhibitory effect of JNU-144 treatment on EMT, we measured the expressions of several regulatory genes in mRNA and protein levels. As shown in Fig. 4a and Figure S4a, the mRNA levels of E-cadherin were significantly increased in a dose- and time-dependent manner, while the mRNA levels of vimentin, N-cadherin, β-catenin and zonula occludens-1 (zo-1) showed no remarkable changes. However, we observed a distinct change after JNU-144 treatment at protein level (Fig. 4b). This suggests that JNU-144 can function in a post-transcriptional way to modulate protein expression. Consistent with previous results, the increase of E-cadherin and the reduction of vimentin and N-cadherin were confirmed by immunofluorescent staining (Fig. 4c). To verify whether the reduction of vimentin and β-catenin protein was caused by protein instability, we used proteasome inhibitor MG-132 and lysosome inhibitor chloroquine and ammonium chloride to pre-treat SMMC-7721 cells followed by JNU-144 treatment. Subsequently, the cell lysates were separated by sodium dodecyl sulfate polyacrylamide gel electrophoresis (SDS-PAGE) and probed with specific antibodies. As we can see, the proteasome inhibitor MG-132 blocked the degradation of β-catenin, while the lysosome inhibitor ammonium chloride dramatically reduced the degradation of vimentin (Fig. 4d). We obtained similar results in HepG2 cells (Figure S4b–d).

### JNU-144 suppresses liver xenograft tumor growth *in vivo*

**Figure 5:**
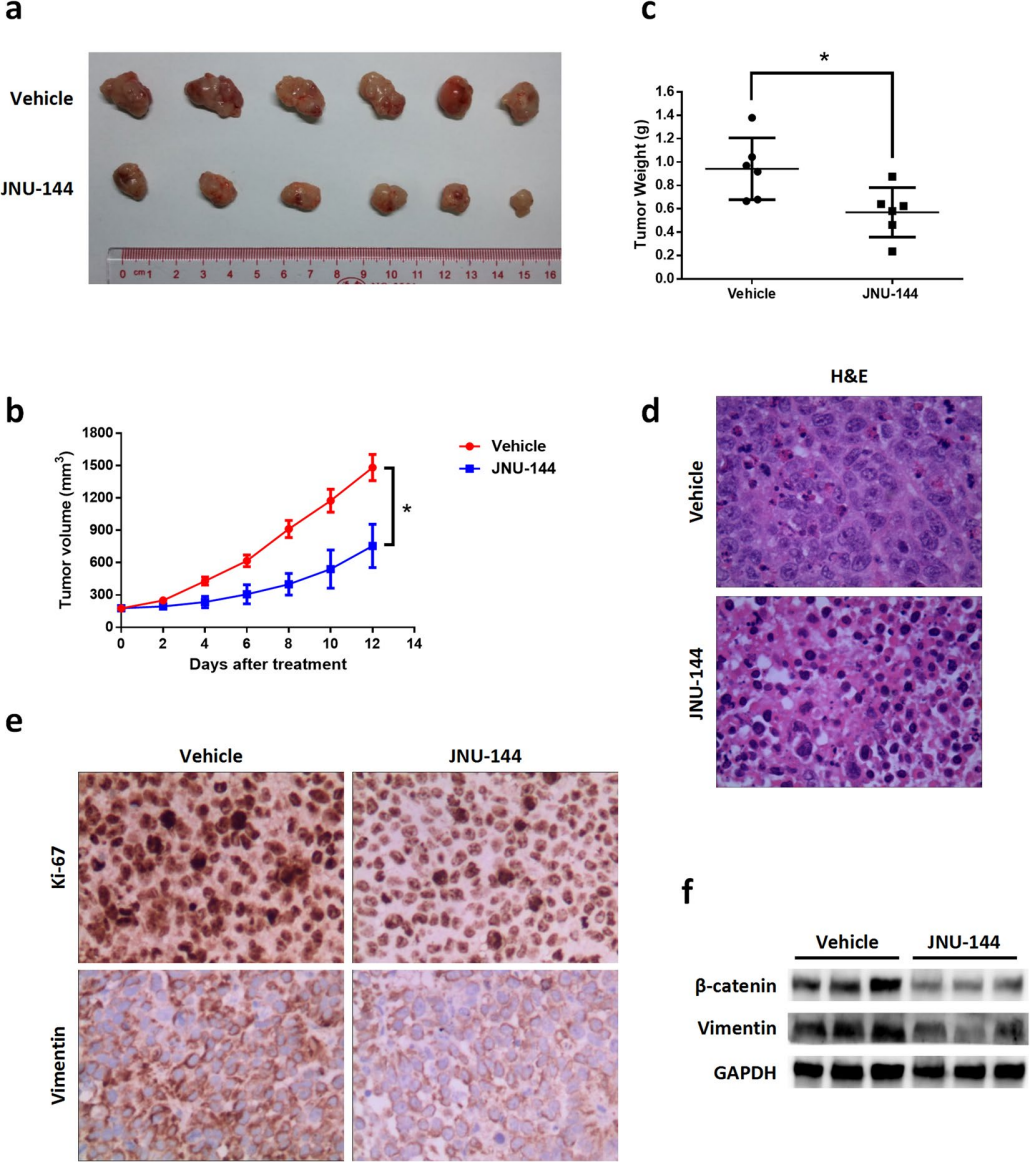
JNU-144 suppresses liver xenograft tumor growth *in vivo*. Nude mice bearing SMMC-7721 xenograft tumors were treated with JNU-144 or vehicle, which was administered by i.p. injection once at 10 mg/kg every two days, six times in total. After implantation, tumor volumes were measured with a slide caliper every two days (**b**). On day 12, the mice were sacrificed and tumors were removed, photographed (**a**) and weighted (**c**). H&E staining (**d**) and immunostaining (**e**) were performed with tumors from vehicle- and JNU-144- treated mice. (**f**) Expression level of relative genes in tumor tissues were detected by immunoblotting. *p < 0.05 compared with the control group.

To determine whether JNU-144 was effective against tumor growth *in vivo*, we injected SMMC-7721 cells subcutaneous injection (s.c.) into nude mice. After implantation, tumor volumes and body weights were measured every two days. When the tumor volumes reached approximately 100 mm^3^, JNU-144 or vehicle was administered to tumor-bearing mice by intraperitoneal (i.p.) injection (10 mg/kg) once every two days, six times in total. The mice were sacrificed after treatment, and tumor tissues were excised, weighed and photographed. As expected, JNU-144 inhibited liver xenograft tumor growth *in vivo* (Fig. 5a–c) without significant host toxicity, which was monitored by changes in body weight and organ abnormalities (Figure S5a,b). Consistently, the H&E staining analyses showed that the tumor tissues of the JNU-144-treated group exhibited decreased cell density and massive cell death characterised by karyopyknosis and nuclei loss (Fig. 5d). To confirm this observation *in vitro*, we evaluated some typical protein levels *in vivo* by immunohistochemistry and western blot analyses. JNU-144 treatment decreased the expression of vimentin and ki-67, a cellular marker for proliferation (Fig. 5e). The results of the western blot analyses were consistent with the observations in hepatoma cells (Fig. 5f). Taken together, these data suggest that JNU-144 treatment suppresses the growth of liver xenograft tumors.

## Discussion

JNU-144 is a novel natural product isolated from *L. erythrorhizon*. In this study, we demonstrated its antitumor activity. Specifically, the anti-cancer mechanism of JNU-144 in hepatoma cells was investigated. We confirmed the ability of JNU-144 to inhibit hepatoma cell viability and proliferation *in vitro* and *in vivo*. JNU-144 treatment inhibits hepatoma cells growth and proliferation by downregulating mTOR activation. The cytotoxic effect of JNU-144 is caused by apoptotic cell death triggered by activation of the intrinsic apoptosis pathway. Furthermore, EMT is suppressed by JNU-144 through reprogramming of the gene expression profile. In conclusion, JNU-144 exerts potent anticancer activity in hepatoma cells and has the potential to be developed as a novel therapeutic drug.

The phosphatidylinositol 3 kinase (PI3K)/AKT/mTOR pathway is an intracellular signaling pathway that is important for numerous cellular functions, including proliferation, survival, migration, invasion and metabolism, and it is constitutively activated in the majority of human cancers^27^. mTOR, a key regulator of protein translation^28^, propagates the signaling from AKT activation and phosphorylates several downstream targets. Given the importance of PI3K/AKT/mTOR signaling in the development of human cancers, the inhibitory function of JNU-144 towards cell growth and proliferation may be attributed to its suppression of mTOR activation. Nevertheless, AKT was activated by JNU-144 treatment, which suggested that the suppression of mTOR has no relation to AKT. On the other hand, we found that JNU-144 treatment inhibited ERK1/2 activation in a dose- and time-dependent manner (Fig. 1g,h and S1e,f), which is consistant with recent reports that revealed AKT independent regulation of mTOR signaling by the ERK/MAPK pathway^20^. These data suggested that JNU-144 induced mTOR inhibition may be mediated by MEK/ERK pathway. The mechanism beneath it requires further study.

Apoptosis plays a crucial role in diverse biological processes, including normal cell turnover^29,30^, embryonic development^31,32^ and immune function^33,34^. Unlike necrosis, apoptosis is a kind of programmed cell death with a different morphology and biochemical mechanism, including cell shrinkage, dynamic membrane blebbing and dependence of caspase cascade activation^35^. To date, studies have shown that there are two main apoptotic pathways: the extrinsic pathway mediated by death receptor and the intrinsic pathway mediated by mitochondria^36,37^. The Bcl-2 family of proteins is mainly responsible for mitochondrial membrane permeability regulation and can be divided into either pro-apoptotic (including bax and bak) or anti-apoptotic (including bcl-2) proteins^38^. We observed morphological changes, such as membrane blebbing, cell shrinkage and nuclear condensation. Moreover, the Annexin V/PI staining indicated that JNU-144 treatment induces apoptosis, which is rescued by z-VAD-fmk, a pan caspase inhibitor (Fig. 2d and S2b). The proteolytic caspase cascade is an important biochemical feature of apoptosis, we succeed to detect the cleavage of pro-caspase 3 after JNU-144 treatment in SMMC-7721 (Fig. 2f,g) and HepG2 cells (Figure S2c). The decrease of bcl-2 and increase of bax and bak expression suggested that this apoptotic cell death might be induced by the intrinsic pathway.

EMT, a process during which epithelial cells transdifferentiate into mesenchymal cells, is deeply involved in cell development^39^, wound healing^40^, stem cell differentiation^41^ and tumor progression^10,42^. Downregulation of E-cadherin and upregulation of N-cadherin, the so called ‘cadherin switch’ that alters cell adhesion, is a hallmark of EMT^43,44^. Our results showed an adverse cadherin switch after JNU-144 treatment, which indicated that EMT was suppressed. Zona occludens protein ZO-1, a peripheral membrane adaptor protein that links junctional transmembrane proteins, is required for tight junction formation and function^45^. Therefore, the induction of ZO-1 expression by JNU-144 may contribute to its inhibitory effect on EMT. Vimentin is a type III intermediate filament protein and is considered a marker of cells undergoing EMT^46^. Transcription factor-8 (TCF8)/human zinc finger E-box-binding homeobox 1 (ZEB-1), a zinc-finger E-box-binding transcription factor, reprograms the gene expression profile that defines the EMT phenotype^47,48^. Nuclear β-catenin upregulates the expression levels of genes supporting tumor invasion^49^, and there is growing evidence indicating that Wnt/β–catenin signaling plays an important role in EMT^50,51^. Consistent with these previous reports, we observed a decrease of these pro-EMT proteins. However, the mRNA levels did not reduce proportionally, which suggests that JNU-144 may enhance their instability. Mammalian cells contain two distinct proteolytic pathways: the lysosome degradation pathway and the ubiquitin-proteasome pathway. Extracellular proteins that enter the cell are degraded in lysosomes^52^. Proteins that are targeted for degradation by the proteasome are tagged with a polyubiquitin chain. Then, the polyubiquitinated protein is degraded by proteasome, a 26 s protease complex^53^. Thus, we pre-treated SMMC-7721 cells with MG-132^54^, a proteasome inhibitor, chloroquine^55^ or ammonium chloride^56^, an inhibitor of lysosome, followed by JNU-144 treatment. As can be seen in Fig. 4d, the decrease of β-catenin and vimentin was blocked by MG-132 and ammonium chloride, respectively, which means that the degradation of β-catenin is proteasome dependent, whereas the degradation of vimentin is lysosome dependent. Quite unexpectedly, chloroquine did not inhibit vimentin degradation, as chloroquine, like ammonium chloride, is a lysosomotropic agent that accumulates inside the lysosome and raises the lysosomal pH, which leads to the inhibition of lysosomal enzymes^57,58^. It is noteworthy that the inhibition of protein degradation caused by ammonia was accompanied by lysosomal vacuolisation^59^. This lysosomal vacuolisation may be connected to the difference between the effects of chloroquine and ammonium chloride on vimentin degradation. The more precise mechanism remains to be elucidated.

## Methods

### Cell lines and reagents

Human hepatoma cell lines SMMC-7721, HepG2, YY-8103, QGY-7703, Huh7 and PLC/PRF/5 were purchased from the American Type Culture Collection (ATCC, Manassas, USA). The cells were cultured in dulbecco’s modified eagle medium (DMEM) supplemented with 10% foetal bovine serum (FBS, Hyclone, Logan, UT, USA), 100 U/mL penicillin and 100 mg/mL streptomycin. All cells were cultured at 37 °C in a 5% CO_2_ incubator. JNU-144 was isolated and identified as described in our previous study^18^. FITC Annexin V Apoptosis Detection Kit (556547) was obtained from BD Biosciences (Singapore). Trypan blue (T8070) and 3-(4,5-Dimethylthiazol-2-yl)-2,5-diphenyltetrazolium bromide (MTT, M8180) were purchased from Solarbio (Beijing, China). Matrigel (356234) and transwell chambers (3422) were obtained from Corning (New York, USA). 4′,6-diamidino-2-phenylindole (DAPI, D9542), crystal violet (C6158), MG-132 (C2211), chloroquine diphosphate salt (C6628) and ammonium chloride (V900222) were purchased from Sigma-Aldrich (St. Louis, MO, USA). TRIzol™ Reagent (15596018) and z-VAD-fmk (tlrl-vad) were obtained from Invivogen (Carlsbad, CA). HiFi-MMLV cDNA Kit (CW0744M) was obtained from Cwbiotech (Beijing, China), Forget-Me-Not™ EvaGreen® qPCR Master Mix (31042-1) was obtained from Biotium (Hayward, California, USA) and the anti-human mTOR (2972), phosphorylated mammalian target of rapamycin (p-mTOR) (2974), AKT (4691), phosphorylated serine/threonine kinase 1 (p-AKT) (4060), extracellular regulated protein kinases (ERK1/2) (4695), phosphorylated extracellular regulated protein kinases (p-ERK1/2) (4370), Caspase-3 (9662), cleaved caspase-3 (9664), Bax (2774), Vimentin (5741), N-cadherin (13116), β-catenin (8480), ZO-1 (8193), TCF8 (3396) and β-actin (3700) were obtained from Cell Signaling Technology (Danvers, MA, USA). The anti-human E-cadherin (MAB-0589) and Bcl-2 (MAB-0014) were obtained from MXB^®^ Biotechnologies (Fuzhou, Fujian, China). Finally, the anti-human GAPDH (60004-1-lg) was obtained from Proteintech (Rosemont, Illinois, USA).

### Cell viability assay

Cell viability was measured with the MTT assay. Briefly, the cells were seeded at a density of 1 × 10^4^ cells/well in a 96-well plate for 24 h, and then the cells were treated with DMSO or different concentrations of JNU-144 for the indicated time. After the exposed period, 100 μL of MTT (5 mg/mL in DMEM) was added to each well for 4 h. Thereafter, the medium containing MTT was removed and 150 μL dimethyl sulfoxide (DMSO) was added to solubilise the formazan crystals. The absorbance (OD) was measured using a spectrophotometric microtiter plate reader at 590 nm.

### Colony formation assay

The cells were harvested, and an appropriate number of cells per well were seeded on a 24-well plate in triplicate. The cells were treated as indicated for 12 h, and then they were collected and seeded on a 12-well plate at a density of 5 × 10^2^ cells/well. The cells were incubated for 10 days. The resulting colonies were stained with 0.4% trypan blue for 30 min and counted by microscopy.

### Western blot analysis

Briefly, cell pellets were lysed in the lysis buffer (20 mM Tris, 150 mM NaCl, 1% triton X-100, 1 mM EDTA, 1 mM EGTA, 0.1% SDS) supplemented with 1 mM phenylmethanesulfonyl fluoride (PMSF). Identical amounts of protein were separated by SDS-PAGE and transferred to a polyvinylidene difluoride membrane. The membrane was probed with the specific primary antibodies after blocking with 5% non-fat milk for 1 h and then with peroxidase-conjugated secondary antibodies. β-actin and glyceraldehyde-3-phosphate dehydrogenase (GAPDH) were used as protein loading controls. The protein of interest was visualised by an immunoblotting chemiluminescence (ECL) reagent.

### Apoptosis assay

Apoptosis assay is performed with FITC Annexin V Apoptosis Detection Kit (556547) according to the manufacturer’s protocol. Briefly, the cells were collected after treatment and wash cells twice with cold PBS and then resuspend cells in 1 × Binding Buffer at a concentration of 1 × 10^6^ cells/mL. Add 5 µL of FITC Annexin V and 5 µL PI, then Gently vortex the cells and incubate for 15 min at RT in the dark. Stained cells were analyzed by flow cytometry and quantified using the CellQuest software. At least 10,000 events were analyzed and compared with control. For DAPI staining, treated cells were fixed in pre-chilled 3.7% methanal for 10 min and then stained with 0.1 μg/mL of 4–6-diamidino-2-phenylindole (DAPI) for 10 min. Nuclei were examined and imaged using a fluorescence microscope after being washed three times.

### RNA extraction and quantitative real-time PCR (qPCR)

Total RNA was extracted with a TRIzol reagent (Invitrogen, USA). cDNA was synthesised using the HiFi-MMLV cDNA Kit (Cwbiotech, China). PCR analyses were performed with Forget-Me-Not™ EvaGreen® qPCR Master Mix (Biotium, USA). The primers used are shown in Supplementary Table S1. The data were normalised to actin expression and further normalised to the negative control. The fold changes were calculated through relative quantification (2^−ΔΔCt^).

### Wound-healing assay

The migratory ability of the SMMC-7721 and HepG2 cells was measured with a wound-healing assay. Cells were seeded at a density of 5 × 10^5^ cells per well on a 12-well plate and grown to about 90% confluence after 24 h. The medium was then removed, and the cells were wounded by manually scraping them with a plastic pipette tip. The cells were washed twice with PBS, and then they were incubated with fresh medium in the presence of JNU-144 or DMSO. To monitor the cell migration, images were captured immediately at 0, 24 and 48 h post wounding with a microscope.

### *In vitro* migration and invasion assay

Cells were treated with DMSO or JNU-144 for 12 h. Subsequently, they were collected and resuspended in serum-free media at a density of 1 × 10^6^/mL and plated at 100 μL cell suspension in chambers with 8 μm pores (Corning, USA). These chambers were put in wells containing fresh media supplemented with 10% FBS. After incubation for 24 h at 37 °C in 5% CO_2_, the cells on the upper surface of the filter were wiped with a cotton swab. Cells that invaded the lower surface of the filter were fixed by 4% paraformaldehyde and stained with 0.1% crystal violet for 30 min. Cell numbers were counted in six random optical fields (×10) per filter under a microscope. A cell invasion assay was conducted in a similar model, except the transwell membrane was pre-coated with 100 μg Matrigel (Corning, USA).

### Immunofluorescent staining

Cells cultured on coverslips were rinsed with PBS and fixed with 4% paraformaldehyde for 10 min after treatment as indicated. The cells were then incubated with PBS containing 0.25% Triton X-100 for membrane permeabilisation and then blocked with 5% bovine serum albumin (BSA) for 1 h. Next, the cells were incubated with specific primary antibodies overnight at 4 °C. Then, the coverslips were washed three times with PBS, followed by incubation for 1 h in the dark with fluorescent secondary antibodies (Invitrogen, USA). Finally, the coverslips were stained with 4-6-diamidino-2-phenylindole (DAPI) for 10 min and captured by a fluorescence microscope.

### Mouse xenograft models

Four- to six-week-old female BALB/c nude mice were obtained from Guangdong Medical Lab Animal Center. To establish the liver tumor xenograft mouse models, 5 × 10^6^ SMMC-7721 cells in 200 μL saline were implanted subcutaneously into the right flank of the nude mice. After implantation, tumor volumes were measured with a slide calliper every 2 days and calculated using the following formula: 0.5 × (length) × (width)^2^. When the tumor size reached 100 mm^3^, the tumor-bearing mice were divided randomly into two groups and treated with the vehicle (1% DMSO in saline) or 10 mg/kg JNU-144 in saline every 2 days by i.p. injection. The mice were sacrificed after six times injection, and tumor tissues were excised, weighed and photographed. Subsequently, xenograft tumor and the major organs were fixed in neutral buffered formalin for western blot analysis, immunohistochemistry and pathological examination.

All of the animals were treated according to protocols approved by Institutional Animal Care and Use Committee of the Shenzhen People’s Hospital. And this study was approved by Institutional Animal Care and Use Committee of the Shenzhen People’s Hospital (Approval Document No. LL-KT-201701017).

### Haematoxylin eosin (H&E) staining and immunohistochemistry

Briefly, fixed tissues and organs were embedded in paraffin. After being cut into 5 mm slices, the sections were stained using a standard H&E procedure. The results were analysed under a phase-contrast Olympus microscope (Olympus America, Inc.). For the immunohistochemical analysis, deparaffinised tumor sections underwent antigen retrieval in sodium citrate buffer (pH 6.0) using the high-pressure method. Then, the slides were blocked with 5% BSA and incubated with specific primary antibodies, followed by incubation with streptavidin–peroxidase horseradish peroxidase conjugated secondary antibodies and stained with a Dako kit (Dako, USA). The sections were counterstained with haematoxylin and analyzed under a phase-contrast Olympus microscope.

### Statistical analysis

Data were analyzed using GraphPad Prism 6.0 software (GraphPad software Inc., USA). The differences between the two groups were estimated with a two-tailed unpaired t-test. The quantitative data are reported as means ± SD from at least three independent experiments. P values less than 0.05 were considered statistically significant.

## Electronic supplementary material

Supplementary information

## Data Availability

The authenticity of this article has been validated by uploading the key data onto the Research Data Deposit public platform (www.researchdata.org.cn), which the Project Title as A new meroterpenoid functions as an anti-tumor agent in hepatoma cells by downregulating mTOR activation and inhibiting EMT.
