# Supplementary material for: A new meroterpenoid functions as an anti-tumor agent in hepatoma cells by downregulating mTOR activation and inhibiting EMT
Source: Sci Rep. 2018 Sep 3;8:13152. doi: 10.1038/s41598-018-31409-2 (PMC6120861; doi:10.1038/s41598-018-31409-2)

## A new meroterpenoid functions as an anti-tumor agent in hepatoma cells by downregulating mTOR activation and inhibiting EMT

Haoqiang Wan^1#^, Jiemei Li^1#^, Keda Zhang^1#^, Xiaoting Zou^1^, Lanlan Ge^1,2^, Fuqiang Zhu^3^, Huirong Zhou^3^, Minna Gong^3^, Tianwa Wang^3^, Dongling Chen^3^, Shusong Peng^3*^, Boping Zhou^1*^, Xiaobin Zeng^1,3*^

^1^Center Lab of Longhua Branch, Shenzhen People’s Hospital, 2nd Clinical Medical College of Jinan University, Shenzhen 518120, Guangdong Province, China.

^2^Department of Infectious disease, Shenzhen People’s Hospital, 2nd Clinical Medical College of Jinan University, Shenzhen 518120, Guangdong Province, China.

^3^Department of pathology (Longhua Branch), Shenzhen People’s Hospital, 2nd Clinical Medical College of Jinan University, Shenzhen 518120, Guangdong Province, China.

^#^These authors have contributed equally to this work.

*Correspondence: [zengxiaobin1983@163.com](mailto:1018yuyang@163.com;); [zhoubp@hotmail.com;](mailto:zhoubp@hotmail.com;) shusongpeng@163.com

**Table S1.** Primers for real-time PCR experiments were shown in table S1.

| Bcl-2-F | 5’-gatgggatcgttgccttatgca-3’ |
| --- | --- |
| Bcl-2-R | 5’-atcacgcggaacacttgattc-3’ |
| Bax-F | 5’-gatgattgccgccgtggaca-3’ |
| Bax-R | 5’-gagtgaggcggtgagcactc-3’ |
| Bak-F | 5’-acgacatcaaccgacgctatg-3’ |
| Bak-R | 5’-cagtgatgcagcatgaagtcg-3’ |
| Vimentin-F | 5’-tgcgtgacgtacgtcagcaa-3’ |
| Vimentin-F | 5’-ccagcagcttcctgtaggtgg-3’ |
| E-cadherin-F | 5’-cagcatcactggccaaggag-3’ |
| E-cadherin-R | 5’-agacctcctgggtgaattcgg-3’ |
| N-cadherin-F | 5’-ctgcaaccgtgtctgttacag-3’ |
| N-cadherin-R | 5’-cgttcctgttccactcatagg-3’ |
| β-catenin-F | 5’-agcagagtgctgaaggtgct-3’ |
| β-catenin-R | 5’-cagcccgaaggacagtacgc-3’ |
| ZO-1-F | 5’-agagatgaacgggctacgct-3’ |
| ZO-1-R | 5’-ggtgtgtgatcatcagcatgc-3’ |
| β-actin-F | 5’-aatcgtgcgtgacattaaggag-3’ |
| β-actin-R | 5’-actgtgttggcgtacaggtctt-3’ |

**Supplementary Figure legends**

**Figure S1.** JNU-144 inhibits cell viability in hepatoma cells by downregulating activation of mTOR. Huh7(**a**), QGY-7703(**b**), YY-8103(**c**) and PLC/PRF/5(**d**) cells were exposed to various concentrations of JNU-144 for 12 h for the MTT assays to evaluate the cell viability. (**e**) HepG2 cells treated with different concentrations of JNU-144 for 12 h were lysed and subjected to immunoblotting for detection of the expression levels of relative proteins. (**f**) HepG2 cells treated with JNU-144 at the concentration of 20 μg/mL for indicated time were lysed and subjected to immunoblotting for detection of the expression level of relative proteins. ***p < 0.001 compared with the control group. Graphs show mean ± SD of triplicate wells and represent three independent experiments.

**Figure S2.** JNU-144 induces apoptosis in hepatoma cells. HepG2 cells stimulated with DMSO or 20 μg/mL JNU-144 in the presence or abcence of z-VAD-fmk (z-VAD), a pan caspase inhibitor which is being widely used as an apoptosis inhibitor, were subjected to colony formation assay (**a**) and apoptosis assay (**b**). (**c**) HepG2 cells stimulated with various concentrations of JNU-144 for 12 h were lysed and subjected to immunoblotting for detection of the expression level of relative proteins.***p < 0.001 compared with the control group; **###**p < 0.001 compared with the JNU-144 treated group. Graphs show mean ± SD of triplicate wells and represent three independent experiments.

**Figure S3.** JNU-144 inhibits EMT in hepatoma cells. (**a**) HepG2 cells were pretreated with DMSO or 10 μg/mL JNU-144 for 12 h, followed by scraping with a pipette tip. The wounded area was photographed after scraping for 0, 24 and 48 h. HepG2 cells pretreated with DMSO or 10 μg/mL JNU-144 for 12 h were used for in vitro migration (**b**) or invasion (**c**) assays. *p < 0.05 compared with the control group; **p < 0.01 compared with the control group. Graphs show mean ± SD of triplicate wells and represent three independent experiments.

**Figure S4.** JNU-144 reprogrammes EMT related gene expression profile. (**a**) Relative mRNA expression levels of EMT related genes of HepG2 cells stimulated with 20 μg/mL JNU-144 for 12 h was detected by real-time PCR. (**b**) HepG2 cells stimulated with various concentrations of JNU-144 for 12 h were lysed and subjected to immunoblotting for detection of the expression levels of relative proteins. (**c**) HepG2 cells stimulated with DMSO or 10 μg/mL JNU-144 for 12 h were immunostained and photographed using a fluorescence microscope. (**d**) HepG2 cells were pretreated with proteasome inhibitor MG-132 (20 μM), lysosome inhibitor ammonium chloride (15 mM) or chloroquine (100 μM) for 12 h, followed by stimulation with DMSO or 20 μg/mL JNU-144 for 12 h. The cells were lysed and subjected to immunoblotting for detection of the expression levels of relative proteins. ***p < 0.001 compared with the control group. Graphs show mean ± SD of triplicate wells and represent three independent experiments.

**Figure S5.** JNU-144 has no significant toxicity in liver xenograft models. (a) Nude mice bearing SMMC-7721 xenograft tumors were treated with JNU-144 or vehicle, which was administered by i.p. injection once at 10 mg/kg every two days, six times in total. After injection, the body weight of the mice was measured every two days. (b) After the mice were sacrificed, the main tissues like heart, liver, spleen, lung and kidney were fixed, and then H&E staining were performed.


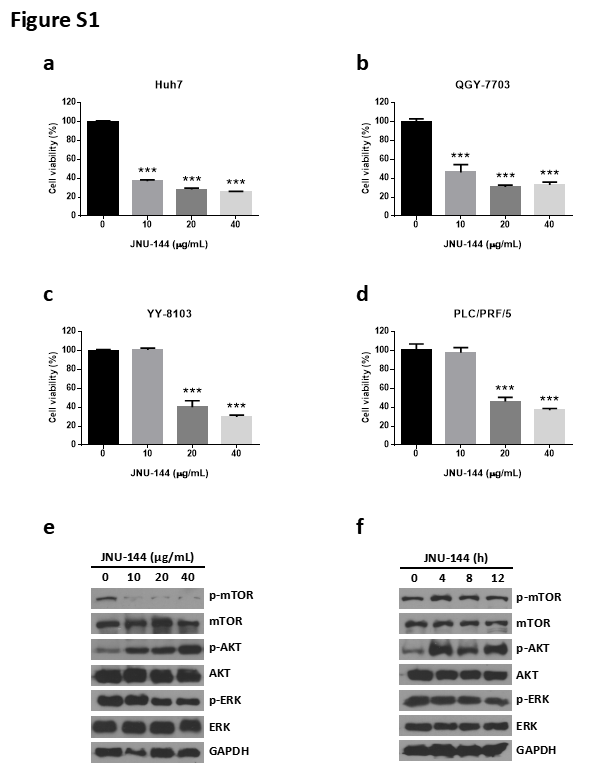


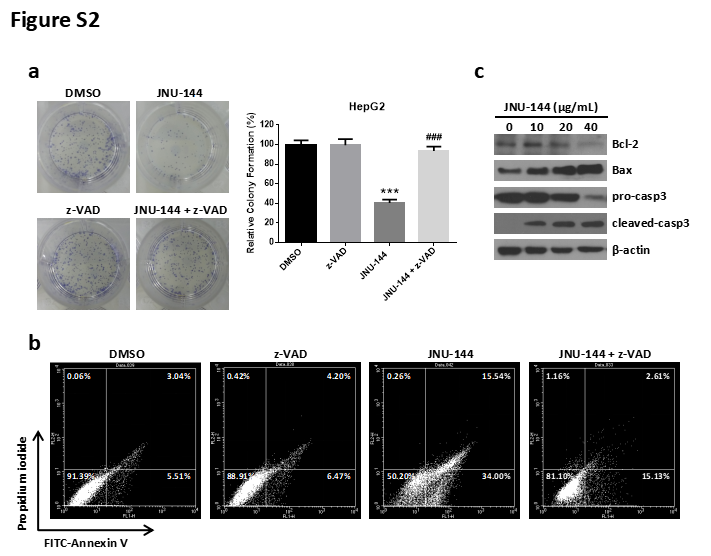


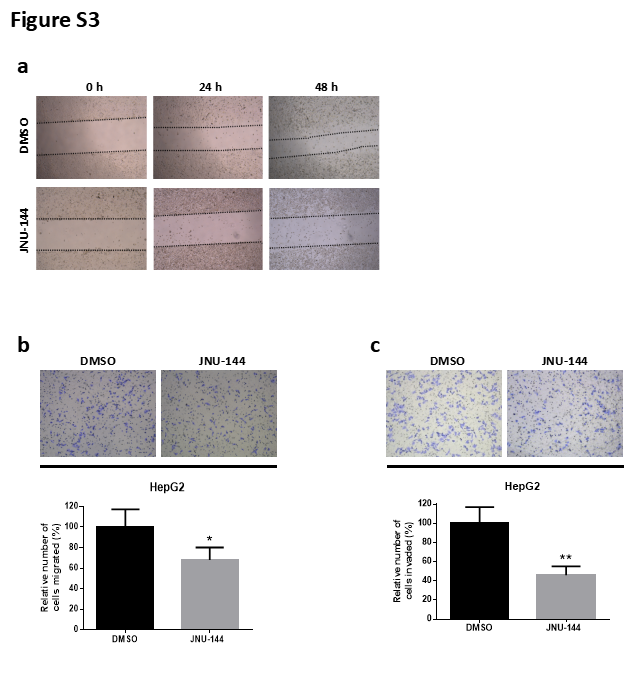


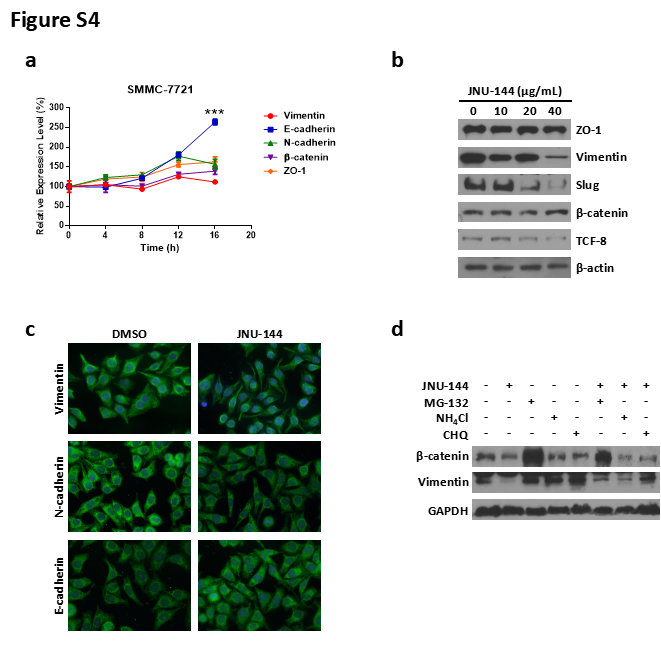


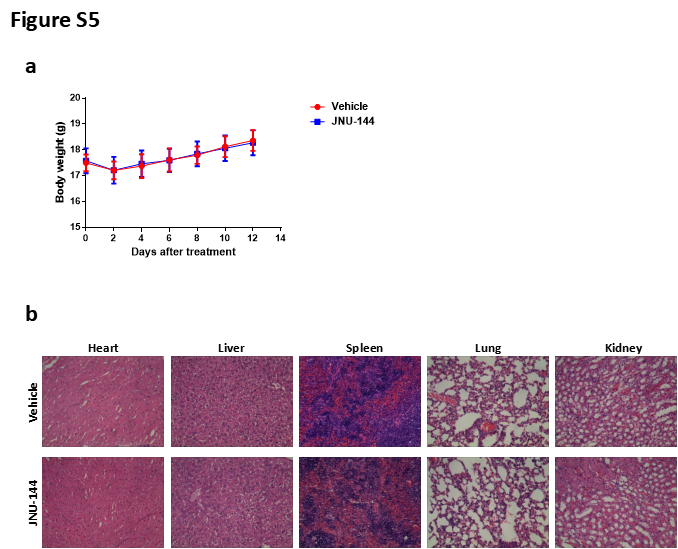

Supplement: Supplementary file 1 — Supplementary information [file 41598_2018_31409_MOESM1_ESM.docx]
